# Exploring the Structure of a School-Based Sexual and Reproductive Health Education Program: A Qualitative Study

**DOI:** 10.5935/1518-0557.20210072

**Published:** 2022

**Authors:** Zahra Bostani Khalesi, Masoomeh Adib, Abdolhosein Emami Sigaroudi

**Affiliations:** 1 Social Determinants of Health Research Center, Guilan University of Medical Sciences, Rasht, Iran; 2 Shahid Beheshti School of Nursing and Midwifery, Guilan University of Medical Sciences,Rasht,Iran; 3 Cardiovascular Diseases Research Center, Department of Cardiology, Heshmat Hospital, School of Medicine, Guilan University of Medical Sciences, Rasht, Iran

**Keywords:** reproductive health, sexual health, education, qualitative study

## Abstract

**Objective:**

Training programs on sexual and reproductive health rank among the most effective strategies to empower individuals and engage in self-care in this field. This study aimed to explore the structure of a school-based sexual and reproductive health education program.

**Methods:**

A qualitative, inductive, content-analysis approach was used in this study. Participants were selected through a purposeful sampling method. Data were collected through individual in-depth interviews with 21 students; 7 key informants; and 3 focus group discussions with parents. Interviews were recorded and transcribed into text manually. Data analysis was carried out using the content analysis method and the MAXQDA11 software.

**Results:**

Three categories of health services emerged from the analysis of participant experiences: 1) empowerment-based education model; 2) optimal educational characteristics; and 3) adolescent-friendly sexual and reproductive health services.

**Conclusions:**

The study found that sexual and reproductive health education programs for adolescent girls should be based on empowerment, desirable educational characteristics, and adolescent-friendly mechanisms.

## INTRODUCTION

Although adolescents form one of the main pillars of our communities, they are regarded as a vulnerable group (World Health Organization, 2021). The main causes of vulnerability are the lifestyle changes and social hazards that individuals in this age range are exposed to (Mosavi *et al*., 2014). Available data indicate that 70% of the deaths in adulthood are linked to behaviors in adolescence (Azzopardi *et al*., 2019).

The institutionalization of health-centered behavior in adolescents has been clearly emphasized in Iran's 1404 Vision document. One of the sections of this document discusses the preservation and promotion of sexual and reproductive health (SRH) (Refaie Shirpak, 2015). SRH may be defined as a person's physical, emotional, and social wellbeing in relation to sexuality and the reproductive system (Abraham *et al*., 2019). Adolescent girls are a vulnerable population facing several serious challenges associated with SRH including sexual abuse, gender inequality, unwanted pregnancy, female circumcision, and sexually transmitted diseases such as HIV/AIDS (Azh *et al*., 2017). Studies in Iran *revealed* that the knowledge *adolescent*s *possess of* SRH is limited (Abedian & Shahhosseini, 2014). Insufficient knowledge about SRH and *lack* of *reliable* access to educational resources call for the institution of sexual and reproductive health education programs, particularly to adolescent girls (MirzaiiNajmabadi *et al*., 2019).

SRH education for adolescent girls is of utmost significance (UNFPA. The Danish Institute for Human Rights, 2014). Most young people go to school. In Iran, 18 million students are currently enrolled in schools across the nation (MirzaiiNajmabadi *et al*., 2019). Therefore, schools are one of the best institutions for conducting health education programs and introducing preventive measures (Lima-Serrano & Lima-Rodríguez, 2014). Schools may be used to implement health promotion programs and help students learn healthy lifestyles (Refaie Shirpak, 2015).

According to the recommendation of the World Health Organization, schools should have a health promotion approach and constantly strengthen their capacity as a healthy environment to live and learn (World Health Organization, 2008). In Iran, formal SRH education programs are not offered in schools, because such educational programs, especially for adolescents, are still taboo (Javadnoori *et al.,* 2018).

Although several studies have been conducted in Iran to determine the educational needs of adolescent girls in SRH (Askari *et al*., 2020; Javadnoori *et al*., 2018; MirzaiiNajmabadi *et al*., 2019), nothing has said about how to organize SRH education programs in schools. The mere assessment of the quantitative needs and views of individuals regardless of their beliefs does not allow the development of an educational structure. Moreover, the study of individual educational viewpoints on SRH is largely based on subjective assumptions and socio-cultural relations, in addition to the framework of values and governing beliefs that dominate this subject. This provides evidence that qualitative research is suitable for investigating this issue. Therefore, qualitative research should be used so that the mechanisms of school-based SRH education are unearthed through interviews with adolescent girls and other stakeholders. Determining the structure of school-based SRH education programs may help high-ranking health policy makers design educational programs to empower female adolescent students and mitigate negative outcomes stemmed from lack of SRH education. The objective of this study was to explore the school-based structure for SRH education.

## MATERIALS AND METHODS

A qualitative, inductive, content analysis approach was used in this study. The purpose of using the content analysis method in this study was to uncover the meanings, priorities, attitudes, and perceptions of adolescent girls in relation to the manner in which SRH education services should be provided based on their verbal messages.

Participants were selected through a purposeful sampling method. *Data were collected through individual in-depth interviews with 21 students* (first and second-year high school girls)*, from seven key informants and three focus group discussions with parents* (five participants in each group). The demographic characteristics of the students are presented in Table 1 and key informant information is described in Table 2. Sampling was continued until we reached the data saturation point. The data saturation point is achieved when the researcher cannot collect new data through more sampling (Maxwell, 2014).

**Table 1.** Demographic characteristics of the students participating in the study

Variables		Number (percentage)		
**Grade**	First-year high school	12 (57.15)		
	Second-year high school	9 (42.85)	Mathematics	3 (33.34)
			Experimental sciences	2 (22.22)
			Humanities	2 (22.22)
			Art	2 (22.22)
**Type of school**	State	16 (76.2)		
	Non-profit	5 (23.8)		
**Residence**	City	18 (85.72)		
	Village	3 (14.28)		

**Table 2.** Demographic characteristics of the key informants participating in the study

N	Gender	Age	Major	Education	Job
1	Female	47	Health education	PhD	Faculty
2	Female	51	Reproductive health	PhD	Faculty
3	Female	33	Midwifery	MSc	Private sector
4	Female	48	Reproductive health	PhD	Faculty
5	Male	55	Educational Specialist	PhD	Faculty
6	Female	53	Health education	PhD	Faculty
7	Female	43	Reproductive health	PhD	Faculty

The participants were selected with maximum diversity in terms of factors such as age, degree and field of study, schools (public and non-profit), and location (city/village). The subjects included were fluent in Persian, resided in Rasht, did not have chronic or mental illnesses, and participated willingly in the study.

The face-to-face interviews were recorded with the permission of the participants and were transcribed word by the word for purposes of analysis. A script was used to guide the interviews. The interview script was prepared based on a literature review and tested in a pilot study. Next is a sample of the questions asked during individual interviews: In your opinion, how important is SRH education for the health of girls? What is your opinion about SRH education for adolescent girls? When you have trouble with puberty issues or have questions about it, what method do you use to find answers?

Each interview lasted between 45-60 minutes. The second interview was performed only after coding the first interview. Data collection continued until the data saturation point was reached. Two focus group discussions were held. Group discussion is a practical method in the needs assessment process, which is performed during or at the end of the program to collect valuable information and determine needs (Denzin & Lincoln, 2015).

After expressing their willingness to join the study, participants were assigned a group based on their preferred time and day to attend the interview sessions. The groups included five first and second-year high school female students. The duration of these sessions was 1.5 hours. After obtaining permission from the participants, the discussions were recorded. The mediator researcher (first author) coordinated and guided the discussions so that the questions in the interview script were covered in the interviews.

During the interviews, the mediator researcher asked questions to the participants, while the observer researcher (second author) supervised the interview conduction process and took notes in special cases for more clarity and to prevent the loss of non-verbal data. The researchers observed ethical considerations pertaining to the participants' rights. The purpose of the study was explained and the privacy of the information, confidentiality, and the right to withdraw from the study at any time were secured.

Formal consent in writing was obtained from all participants. Then, the study was explained to the participants in a class. The subjects willing to join the study were asked to sign an informed consent term. The data were analyzed using the conventional content analysis method with the aid of the MAXQDA_11_ statistical package. In qualitative content analysis, the Graneheim and Lundman's method was used, in which each interview was transcribed in its entirety immediately after they were held, the transcriptions of the interviews were read to confirm the general understanding of their contents, meaning units and primary codes were determined, similar primary categories were categorized into more comprehensive classes, and the latent content in the data for qualitative analysis was determined (Graneheim & Lundman, 2004). Therefore, in this study, immediately after each interview, the text of the interviews was manually transcribed and then typed. The manually transcribed text was reviewed several times, and the primary codes were extracted. Then, the related primary codes were clustered and sorted into categories based on similarities. Finally, the themes were formulated.

In order to analyze the data of this study, Guba and Lincoln's (1989) criteria of credibility, dependability, confirmability and transferability were used (Guba & Lincoln, 1989). This was achieved through constant engagement with the participants, immersion in the data, verification of the results by the participants, reviews by the supervisors, and maximum diversity in the participants. The transferability of the data was achieved by checking the findings with adolescent girls who met the inclusion criteria and participated in the study. To increase data transferability, the setting and culture, specifications of the participants, data collection methods, the method of analyzing the data along with examples of participant statements were prepared to help others have access to the measures taken in the study and characteristics of the population of the study.

To increase the consistency of the findings, codes and the extracted categories were handed to two professors specialized in qualitative research who observed all steps of the study. There was a high level of agreement in the opinions of the two supervisors about the data analysis. In order to check the confirmability of the findings, we benefitted from the viewpoints of experienced professors in the field of SRH who were outside the research team in all stages of the study, including sampling, data collection, analysis and interpretation. In addition, in order to enhance dependability, the researchers tried not to contaminate the process of data collection and analysis with their assumptions.

## RESULTS

A total of 21 female students aged 18-12 years with a mean age of 15±3.2 years were included in the study. The demographic characteristics of the students are given in Table 1.

The key informants in this study were an *Educational Specialist,* health education specialists, SRH specialists, and senior midwifery experts, whose demographic characteristics are given in Table 2.

Three categories of health services emerged from the analysis of participant experiences: 1) empowerment-based education model (with two subcategories: needs-based education model; and theory-based education); 2) optimal educational characteristics (with three subcategories: educational content; educational methods, and trainer characteristics); and 3) adolescent-friendly SRH services (with three subcategories: time characteristics; location characteristics; and service provider characteristics).

The extracted codes and categories were used to explain the participants' understanding of the provision of school-based SRH education services (Table 3).

**Table 3.** Categories and subcategories emerged from the analysis of adolescent girls’ experiences of needs for and manner of school-based SRH education services

Category	Subcategory
Empowerment based education model	Needs-based education
	Model and theory-based education
Optimal educational characteristics	Educational content
	Educational methods
	Trainer characteristics
Adolescent-friendly SRH services	Time characteristics
	Location characteristics
	Service provider characteristics

### 1. Empowerment-based education model

#### 1.1. Needs-based education

The subcategory of needs-based education was extracted from the codes: gender, age, values and beliefs.

#### Gender

*"Girls are more in need of* SRH *education as they have menstruation and become mothers in the future. Girls should receive more attention." (Participant 5)*

#### Age

"Student age should be considered when it comes to education; some issues and concepts might be self-evident for us, but they might be challenging for a 10-12-year-old student." (discussion group, Participant 6)

#### Beliefs and values

*"Student values and beliefs deserve attention. Some families are strict on account of religious issues and students must follow them." (Participant 1)*

### 1.2. Model and theory-based education

Education based on models of health education include two models: the health belief model and the behavioral intention model.

#### Health belief model

*"Using the health belief model in* SRH *education helps adolescents make the right decisions." (Key Informant 2)*

#### Behavioral intention model

*"Behavioral intention model is necessary in effective* SRH *education." (Key Informant 4)*

*"I myself learned a lot in the discussions we had today. It is amazing when an issue is raised and a solution for it is found. There are many unanswered questions that might be raised in these discussions, and an answer is given to these questions." (Key Informant 7)*

### 2.1. Educational content

The subcategory of educational content comprised the five codes: the physiology of the reproductive system; the anatomy of the reproductive system; physical and psychological changes in puberty; high-risk behaviors; and menstruation period care.

#### The anatomy of the reproductive system

*"I, as a 16-year-old girl, am not familiarized with my body's anatomy; I still search for information pertaining to my body in the Internet." (group discussion, Participant 3)*

#### The physiology of the reproductive system

*"I do not know why I should experience menstruation; it is so bad; why do I have to suffer with pain all the time?" (Participant 4, group discussion)*

#### Physical and psychological changes in puberty

*"It would be so informative if puberty were explained to girls aged 11 or 12. They should be informed of what happens to their bodies." (Participant 2)*

#### High-risk behaviors

"I have read in many publications that AIDS is transmitted through high risk sexual behaviors. However, I was never explained what high-risk behaviors are." (Group discussion, Participant 5)

#### Menstruation period care

*"There are many rumors about the menstruation period; sometimes, we might believe them............. For example, lots of people say that you should not take a shower during this time." (Participant 1, discussion group)*

### 2.2. Educational methods

The subcategory of educational methods comprised the following codes: group discussion, brainstorming, teaching with mobile phones, and peer education

#### Group discussion

"When an issue is discussed in a group and the students express their ideas, the students think more about it and become aware of others' ideas. "(Group discussion, Participant 8)

#### Brainstorming

*"In my opinion, this is the best possible method we can use now, sitting next to each other and expressing our ideas. Then you draw conclusions based on the ideas." (Discussion group, Participant 9)*

#### Peer education

*"Education is the best methods for learning." (Key Informant, 2)*

#### Teaching with mobile phones

"In my opinion, teaching with mobile phones is the best possible method, since it is accessible, free, and everyone has one." (Participant 5)

### 2.3. Trainer (teacher) characteristics

The subcategory of trainer characteristics comprised the following codes: adequate knowledge, trustworthiness, and avoidance from prejudice and bias

#### Adequate knowledge

"Academically, he/she should be fine,....should know everything, ...should not only memorize. When one asks a question, he/she should be able to answer it correctly...do not avoid answering correctly." (Discussion group, Participant 7)

#### Trustworthiness

"A person who teaches (trains) should be able to establish a relation with the students, attract their attention, and they should trust him." (Participant 3)

*"Speaking about this issue is really hard, a person who teaches in this area should be trustworthy; there should be a sense of trustworthiness as we ask our questions." (Participant 7)*

#### Avoidance from prejudice and bias

*"In addition to being informed in the subject, the teacher should be trustworthy and reliable form the perspective of the students; she/he should not share with others or report the issues discussed in class." (Key Informant 1)*

### 3. Adolescent-friendly SRH services

#### 3.1. Time characteristics

The subcategory of time characteristics comprised two codes: time of education; and duration of education

#### Time of education

"Time of education and counseling should not necessarily coincide with the time of the educational activities at school. It should be flexible so that students have options." (Key Informant 6)

#### Duration of education

*"The duration of any education is also of utmost importance and should be considered in the planning of the educational program. It should be neither too long - and thus boring - nor too short to the point of affecting the students' understanding." (Key Informant 5)*

### 3.2. Location characteristics

The subcategory of location characteristics consisted of specific physical space and sense of security.

#### Specific physical space

"The place of education should meet the required standards. The best possible way is to designate a classroom or room as the counseling room. It is much better if health care providers organize regular education programs in the counseling room for different groups of students, so that going to the counseling room is not viewed as something taboo." (Key Informant 4)

#### Sense (feeling) of security

*"An adolescent should feel safe and secure when she comes for counseling, she should feel comfortable." (Key Informant 3)*

### 3.3. Service provider characteristics

The subcategory of service provider characteristics comprised two codes: appropriate conduct characteristics; and adequate knowledge of adolescents.

#### Appropriate conduct characteristics

"The individuals working with the adolescents should be well-humored, patient, sociable and trustworthy." (Participant 11)

#### Adequate knowledge of adolescents

*"Having adequate knowledge of adolescents' characteristics and their behavioral changes is necessary for all involved with them." (Key Informant 7)*

## DISCUSSION

The findings of this study showed that almost all participants believed education should be based on needs. This subcategory was extracted from the gender, age, attitudes, and beliefs codes. Some of the participants believed that taking gender into account was essential in SRH education. They believed that the planners and the authorities should plan more SRH education programs for girls with respect to the future role girls have as mothers. In this regard, the United Nations Population Fund warns that ignoring the health needs of teenagers, especially adolescent girls, has caused them to be deprived of proper and appropriate health information and caused irreparable damage (Askari *et al*., 2020). The education of adolescent girls gains momentum since the health of girls is the key to breaking down the poverty cycle between generations and achieving Millennium Development Goals of the World Health Organization (MirzaiiNajmabadi et al., 2019).

In the present study, some of the participants hinted at age-tailored education encompassing individual values and beliefs. SRH education tailored in accordance with age, information and skill needs, and cultural and religious values is necessary to empower students in maintaining and promoting SRH (Mosavi *et al*., 2014).

The majority of the key informants suggested the use of models and theories to increase the effectiveness of education. The results of a study by Havaei *et al*. (2019) showed that the use of education based on protection motivation theory improved the self-care of adolescents in the field of SRH.

Some of the participants believed that the anatomy and physiology of genital organs should be seen as an essential element in SRH education. They believed inadequate knowledge of the anatomy of reproductive organs might promote conditions that cause damage to these organs and endanger the health of individuals. Thoughts and beliefs about sexual organs can affect the sexual behavior of individuals and severely affect the sexual response of individuals in terms of desire, arousal, and orgasm (Crawford *et al*., 1993).

The findings of this study revealed that *half* or *more than half* of the adolescents viewed that addressing the physical and psychological changes in puberty is required in SRH education. Mosavi *et al*. (2014) concluded that in puberty education to adolescents, discussing the physical and mental changes that occur in this period is necessary.

In this study, all participants emphasized the necessity of teaching about high-risk behaviors. High-risk sexual behavior is one of the most susceptible factors for sexually transmitted diseases and increases the probability of physical, psychological, and social negative outcomes for the person (Ramezankhani *et al*., 2011).

There was also an emphasis on the provision of sufficient education about menstrual care by a large number of participants. Menstruation during adolescence is the most important developmental event of a girl's reproductive system. Having sufficient knowledge about the care required in this period is necessary to prevent infection (Salam *et al*., 2016).

In the subcategory of "educational methods", the use of discussion methods, brainstorming, education via mobile phones, and peer education were described as necessary by the participants. The participants expressed that education along with the discussion, brainstorming, and exchange of ideas had a significant role in the effectiveness of SRH education. Brainstorming with the opportunity to discuss and exchange ideas in the process of education not only increases interest in students, but also increases the effectiveness of the educational program and encourages student active participation (Mason-Jones *et al*., 2012).

Peer education was described by most participants as one of the most effective educational methods for the promotion of SRH. Abdi & Simbar concluded that the best method of education is to enrich the relationship between adolescents and trainers through peer education (Abdi & Simbar, 2013).

In the subcategory of "e-learning" and electronic teaching, the participants emphasized the need to develop education through mobile phones. A large number of participants have access to mobile phones and stressed the need to use this tool to teach reproductive health. One of the best methods of virtual learning is to use mobile educational applications (Sadeghi & Heshmati, 2019).

A large number of participants viewed adequate knowledge about SRH as a requirement for the trainers and teachers of health-related topics. A number of participants mentioned a trustworthy trainer or teacher as an absolute necessity in SRH education. Ramezankhani *et al*. (2011) showed that healthcare trainers and teachers are the most important staff that can influence the quality of educational services and student satisfaction, since the quality of such services from the students' perspective is connected mainly to the assessment they make of staff behavior and activities. If the teaching staff lacks the knowledge needed to carry out their tasks and if they fail to treat students properly, students will be dissatisfied.

In the subcategory of "time characteristics", participants raised the time and duration of the educational sessions. Offering flexible hours was noted as the most important time characteristic affecting education and counseling in SRH by a number of participants. The interference of education hours and counseling with class hours is one of the organizational challenges of educational programs in the area of health (Nabe-Nielsen *et al*., 2015).

In the subcategory of "location characteristics", participants stressed the need for having a specific physical space and a sense of security. Having a specific physical space was considered by a number of participants as a significant necessity for holding SRH education. Paying attention to the specific place of education compatible with the psychological and intellectual characteristics of the learners is necessary to achieve educational goals. One of the notable points, which should be considered when choosing the location of education, is that it should provide a noise-free environment (Deschesnes *et al*., 2003).

## CONCLUSION

The findings of this study showed that the structure of SRH education for adolescent girls should be based on empowerment, desirable educational characteristics, and adolescent-friendly mechanisms. However, in qualitative studies, the findings depended to a large extent on the cultural and social aspects of the data. Sampling with maximum diversity in terms of age, education, and different levels of economic and social status are among the strengths of the present study. Given the existing cultural resistance to sexual issues in society, the establishment of trust and close relationships between researchers and participants was another strength of this study. As the sampling was done in the city of Rasht and as the formation of sexual schemas may vary even in the same society from city to city, as in other qualitative studies, the non-generalizability of findings is one of the limitations of this study.
